# Current taxonomy of phages infecting lactic acid bacteria

**DOI:** 10.3389/fmicb.2014.00007

**Published:** 2014-01-24

**Authors:** Jennifer Mahony, Douwe van Sinderen

**Affiliations:** ^1^Department of Microbiology, University College CorkCork, Ireland; ^2^Alimentary Pharmabiotic Centre, Biosciences Institute, University College CorkCork, Ireland

**Keywords:** *Lactococcus*, *Streptococcus*, *Lactobacillus*, dairy, food fermentation, genetics

## Abstract

Phages infecting lactic acid bacteria have been the focus of significant research attention over the past three decades. Through the isolation and characterization of hundreds of phage isolates, it has been possible to classify phages of the dairy starter and adjunct bacteria *Lactococus lactis, Streptococcus thermophilus, Leuconostoc* spp., and *Lactobacillus* spp. Among these, phages of *L. lactis* have been most thoroughly scrutinized and serve as an excellent model system to address issues that arise when attempting taxonomic classification of phages infecting other LAB species. Here, we present an overview of the current taxonomy of phages infecting LAB genera of industrial significance, the methods employed in these taxonomic efforts and how these may be employed for the taxonomy of phages of currently underrepresented and emerging phage species.

## Introduction

The lactic acid bacteria (LAB) are a heterogeneous group of Gram positive, non-spore-forming bacteria with a rod-shaped or coccoid morphology. As their name suggests, lactic acid is the predominant end-product when LAB engage in hexose fermentation, and it is due to the pre-servative and palatable properties of lactic acid that has for many centuries rendered this group of bacteria applicable in food and feed fermentations, in particular for the production of dairy products. Strains of *Lactococcus lactis* and *Streptococcus thermophilus* are the most intensely employed starter bacteria in the dairy fermentation industry globally (Deveau et al., 2006), while strains of *Lactobacillus* spp. and *Leuconostoc* spp. are widely used as adjuncts in such processes (Nieto-Arribas et al., 2010). Furthermore, in vegetable fermentations, ecological studies have reported the complex and evolving microbial landscape with strains of *Lactobacillus*, *Pediococcus, Leuconostoc* and *Weisella* spp. implicated at various stages of the fermentation (Lu et al., 2003, 2012). However, as with most living organisms, LAB are susceptible to viral infection by (bacterio) phages, which may impact on the quality, flavor and texture of the final product. The application of these bacteria in modern fermentation processes involves intensive production and throughput, thereby increasing the risk of bacteriophage infection. Phages are particularly problematic in fermentation systems that repeatedly use the same cultures or culture mixes/rotations as phages are known to persist in the processing environs until a suitable host is available to infect. Consequently, phages of LAB have enjoyed significant attention, particularly over the past three decades. All LAB-infecting phages belong *Caudovirales* order and most of them to the *Siphoviridae* family that possess long non-contractile tails and isometric or prolate capsids (Mahony et al., 2012a) Additionally, phages with short non-contractile tails (*Podoviridae*) and those displaying long contractile tails (*Myoviridae*) have also been described for some LAB genera (Chibani-Chennoufi et al., 2004; Chopin et al., 2007; Deasy et al., 2011). Undoubtedly, the most intensely researched LAB-infecting phages are those of the dairy starter bacteria *L. lactis* and *S. thermophilus* (Neve et al., 1998; Lucchini et al., 1999; Quiberoni et al., 2000; Brussow and Desiere, 2001; Proux et al., 2002; Mahony et al., 2006; Guglielmotti et al., 2009; Rousseau and Moineau, 2009; Collins et al., 2013). In recent years, genome sequencing technologies have improved and diversified drastically, and this has probably been the single greatest driving force behind the acquisition of current data regarding LAB-infecting phage biodiversity, taxonomy and evolution. Current phage taxonomic efforts significantly depend on comparative genomic analysis and derived information. Phage taxonomy is a contentious issue, yet a highly important one since such classifications are core to the development of detection tools and prevention and control measures. Here, we will review the changing face of LAB phage taxonomy, the major advances to date and how such taxonomic efforts may influence future efforts at minimizing the risk of phage infection.

### Lactococcal phages

Phages that infect host strains with resident prophages and/or phage-resistance systems are subject to significant genome rearrangements, which appears to be a major evolutionary driving force among such phages (Labrie and Moineau, 2007). Therefore, it is of great significance that the genome sequences of a number of lactococcal strains and their resident prophages have become available to understand the dynamic processes that may lead to such genome rearrangements (Chopin et al., 2001; Ventura et al., 2007; Wegmann et al., 2007; Siezen et al., 2010; Ainsworth et al., 2013; Du et al., 2013). *L. lactis* strains employed in the dairy industry belong to one of two subspecies, namely *L. lactis* ssp. *lactis* or *L. lactis* ssp. *cremoris*. While lactococcal strain diversity may be limited, their infecting phages have proven their genomic elasticity and evolutionary capabilities in order to survive and evade hygiene measures, processing conditions and host-encoded phage-resistance mechanisms (McGrath et al., 1999; Scaltriti et al., 2010; Samson et al., 2013). To a large degree, this co-evolution, coupled to the intensity of production, has supported the ever-increasing genetic diversity of these phages as we currently recognize and classify them.

Phages of *L. lactis* were first classified in 1984 into four groups based on morphology, serological reactions and DNA-DNA hybridization of 25 phages (Jarvis, 1984). This study was the basis of further classifications of lactococcal phages resulting in the identification of dominant species in isolation studies and furthermore the identification of rarely encountered and emerging species (Prevots et al., 1990).

In 1991, this classification was updated and 12 species were identified based on DNA homology and morphology (Jarvis et al., 1991). The virion morphologies were identified as belonging to one of two families i.e., *Siphoviridae* and *Podoviridae*. In 2002, the lactococcal phage BK5-t was proven to be a member of the polythetic P335 species, which has both lytic and temperate members (Labrie and Moineau, 2002), thus reducing the number of lactococcal phage species to eleven.

Most recently, in 2006, Deveau and colleagues reassessed existing phage isolates of *L. lactis* and reduced the number of currently existing lactococcal phage species to ten (Deveau et al., 2006). This re-classification highlighted the extinction of the P107 species and the amalgamation of BK5-t, 1483 and T187 in the P335 species (Deveau et al., 2006). Furthermore, it also highlighted the emergence of new species, such as the Q54 and 1706 species, which were previously unknown or unclassified (Deveau et al., 2006). Over the past decade, representative members of the rare and emerging lactococcal phage species, 949 (Samson and Moineau, 2010), P087 (Villion et al., 2009), P034 (Kotsonis et al., 2008), Q54 (Fortier et al., 2006), 1358 (Dupuis and Moineau, 2010), KSY1 (Chopin et al., 2007) and 1706 species (Garneau et al., 2008), have been sequenced and providing essential information to corroborate this classification scheme.

The above taxonomic studies have all compounded the necessity of combining taxonomic methods (including electron microscopy, DNA-DNA hybridizations/genome sequencing) that complement each other and provide an effective means for grouping phages (Jarvis, 1984; Jarvis et al., 1991; Deveau et al., 2006). In 1990, Prevots and colleagues identified that the virulent 936 species dominated their collection of 101 phage isolates (Prevots et al., 1990) and from information gathered over the ensuing 23 years, this dominance has been retained (Deveau et al., 2006; Rousseau and Moineau, 2009; Castro-Nallar et al., 2012; Murphy et al., 2013). The genome architecture and content of the 936 phages is highly conserved and the success of this species may be attributed to the limited number of strains available to the dairy industry, permitting their propagation and evolution (Mahony et al., 2012b). The P335 phage species is currently the second most frequently isolated species in the dairy industry and this represents a genetically diverse group of phages that may be lytic or temperate (Mahony et al., 2013). Correlating with their industrial significance, the 936 and P335 species phages also dominate in terms of fundamental research pertaining to their genomics and phage-host interactions and serve as models for phages of a variety of Gram positive bacterial hosts (Veesler et al., 2012; Bebeacua et al., 2013; Collins et al., 2013). To date, in excess of 70 lactococcal phage genomes have been sequenced to completion with approximately 70% of these belonging to the 936 species according to the EMBL-EBI website at the time of writing (www.ebi.ac.uk/genomes/phage.html) (Table 1). Given the lack of complexity of most lactococcal starter cultures, it is not surprising that these species continue to dominate and evolve, however, there are ample possibilities for genetic rearrangements and development of novel species as has been observed in the case of the Q54 species (Fortier et al., 2006). The emergence of such novel species highlights the necessity of regular revisions of the taxonomy of these phages. Furthermore, while the small, isometric-headed phages are the most abundant morphotype of lactococcal phages, the observation of lactococcal siphophages with unusually long tails (949 species), or *Podoviridae* with decorated capsid structures (KSY1), indicates that morphological assessment remains a useful tool in the taxonomic characterization of such phages as a complement to genotyping.

**Table 1:** **Current taxonomy of LAB phages with sequenced members**.

**Host**	**Phage family**	**Phage species**	**No. of fully sequenced members**	**Taxonomy reference(s)**
*L. lactis*	*Siphoviridae*	936	51	Deveau et al., 2006
		P335	15	Deveau et al., 2006
		c2	2	Deveau et al., 2006
		1358	1	Deveau et al., 2006
		Q54	1	Deveau et al., 2006
		P087	1	Deveau et al., 2006
		1706	1	Deveau et al., 2006
		949	2	Deveau et al., 2006
	*Podoviridae*	P034	1	Deveau et al., 2006
		KSY1	1	Deveau et al., 2006
*S. thermophilus*	*Siphoviridae*	*cos*	6	Le Marrec et al., 1997
		*pac*	6	Le Marrec et al., 1997
		5093-like	1	Mills et al., 2011
*Ln. mesenteroides*	*Siphoviridae*	Group Ia and b	2	Ali et al., 2013
*Ln. peudomesenteroides*	*Siphoviridae*	Group IIa–d	2	Ali et al., 2013
*Lb. brevis*	*Myoviridae*	Unnamed	1	Deasy et al., 2011; Jang et al., 2011
*Lb. casei*	*Siphoviridae*	Unnamed	1	Villion and Moineau, 2009
*Lb. delbrueckii*	*Siphoviridae*	Unnamed	6	Villion and Moineau, 2009
*Lb. fermentum*	*Siphoviridae*	Unnamed	2	Yoon and Chang, 2011; Zhang et al., 2011
*Lb. gasseri*	*Siphoviridae*	Unnamed	1	Villion and Moineau, 2009
	*Myoviridae*	Unnamed	1	Villion and Moineau, 2009
*Lb. helveticus*	*Myoviridae*	Unnamed	1	Zago et al., 2013
*Lb. paracasei*	*Siphoviridae*	Unnamed	2	Villion and Moineau, 2009
	*Myoviridae*	Unnamed	1	Alemayehu et al., 2009
*Lb. plantarum*	*Siphoviridae*	Unnamed	5	Villion and Moineau, 2009
	*Myoviridae*	Unnamed	1	Villion and Moineau, 2009
*Lb. rhamnosus*	*Siphoviridae*	Unnamed	1	Villion and Moineau, 2009
*Lb. sanfranciscensis*	*Siphoviridae*	Unnamed	1	Ehrmann et al., 2013

### *S. thermophilus* phages

In contrast to the phages of *L. lactis*, all phages infecting *S. thermophilus* display a similar morphology with long, non-contractile tails (typically more than 200 nm in length) and isometric capsid structures, thus belonging to the *Siphoviridae* family (Brussow et al., 1994; Bruttin et al., 1997; Levesque et al., 2005; Guglielmotti et al., 2009; Zinno et al., 2010; Mills et al., 2011). Therefore, electron microscopy and associated morphological analysis provides little scope for differentiation between these phages, thus necessitating the application of other methods of discernment.

In 1994, host range and serological reaction analysis of 81 phages infecting *S. thermophilus* directed the first significant classification of these phages into four classes (Brussow et al., 1994). Only a few years later in 1997, a refinement of this classification was determined through the combined application of DNA restriction profiling, structural protein profiling and host range analysis defined that these phages should be classified into two major groups (Le Marrec et al., 1997). These groups were accordingly named the *cos* (cohesive ends) and *pac* (headful packaging method) groups, in congruence with their mode of DNA packaging. This taxonomic system was upheld until the recent isolation of phage 5093, which infects the Mozzarella starter strain CSK939 (Mills et al., 2011). The genome of this phage was sequenced and revealed a novel genotype among *S. thermophilus* phages. It possesses greater homology to non-dairy streptococcal prophage sequences than to the genomes of sequenced *S. thermophilus* phages. This singular phage represents the newest addition to the lactic streptococcal phage taxonomic grouping system and, as yet, remains the only known member of this third species of *S. thermophilus* phages (Table 1). Furthermore, morphological analysis of this phage revealed globular structures at the tail tip region, a novel feature among lactic streptococcal phages, again reinforcing the application of morphological assessment of phage isolates in parallel with other characterization tools.

### *Leuconostoc* phages

*Leuconostoc* spp. are part of undefined composite starter mixes of many semi-hard cheeses and are required for aroma and flavor formation in such cheeses (Cogan and Jordan, 1994). Phages of *Leuconostoc* spp. have received growing and deserved attention in recent years in terms of phage isolation studies and genomic analysis pertaining to vegetable and dairy fermentations (Sutherland et al., 1994; Gindreau et al., 1997; Greer et al., 2007; Lu et al., 2010; Kleppen et al., 2012; Ali et al., 2013; Kot et al., 2013). With respect to those infecting dairy starter and adjunct strains of *Leuconostoc mesenteroides* and *pseudomesenteroides*, the most significant taxonomic classification has been provided this year following the analysis of 83 phages by host range, morphology and DNA homology (Ali et al., 2013). This resulted in the identification of species-specific groups capable of infecting one species of *Leuconostoc* (Table 1). The phages were primarily grouped into two major classes based on their non-overlapping host ranges, I and II (i.e., those capable of infecting either *Ln. mesenteroides* or *Ln. pseudomesenteroides* strains). All phages were observed to possess long non-contractile tails and isometric capsids, consistent with the features of *Siphoviridae* phages but with distinct baseplate appendages at their tail tip regions. In the case of *Ln. mesenteroides* (group I), one dominant species of phages with globular appendages (15 of 16 phages assessed) classified as species Ia, while a second species Ib is represented by a single isolate that did not display the globular appendages in its baseplate, but was shown to contain extended Y-shaped appendages (Ali et al., 2013). Phages capable of infecting *Ln. pseudomesenteroides* (group II) are grouped into four sub-groups and all present with a smaller baseplate structure than their *Ln. mesenteroides*-infecting counterparts (25 nm vs. 40 nm). Phages possessing a distinct collar structure below the phage head were classified as group IIa, while those without a collar were termed members of group IIb. A third group, IIc, is composed of isolates presenting with a “fluffy” baseplate appendage while the fourth group, IId, contains members that display unusual striations in the phage tail (Ali et al., 2013). In contrast to *Ln. mesenteroides* and *pseudomesenteroides*, phages infecting *Leuconostoc lactis* are rarely reported, representing a major knowledge gap in terms of the overall taxonomy of dairy *Leuconostoc* phages (Johansen and Kibenich, 1992), however, this underrepresentation may be due to the relatively low levels of usage of strains of this species in dairy fermentations (Zamfir et al., 2006). The morphological diversity of phages infecting *Leuconostoc* species is quite striking given the limited number of strains that are available in the dairy setting. Considering the important role of *Leuconostoc* strains in flavor and aroma development in many fermented dairy products, this may represent an interesting and emerging area of LAB phage research. The isolation and characterization of further phages and of the dominant species as well as those of *Ln. lactis* would permit the development of further classification schemes and increasingly sophisticated detection tools for *Leuconostoc* phages, perhaps allowing a correlation to be made between phage prevalence and flavor development (or lack/reduction thereof), thus revealing the exact role of *Leuconostoc* strains within a given fermentation.

### *Lactobacillus* phages

*Lactobacillus* species are widely used as starter and adjunct cultures for certain food fermentations including the production of yoghurt, cheese, sauerkraut, pickles, and, in conjunction with yeasts, sourdough (Lu et al., 2003; Foschino et al., 2005). Some are used in the dairy industry for their purported probiotic effects (Felis and Dellaglio, 2007). In addition to these food fermentation uses of lactobacilli, some species are associated with food spoilage, e.g., *Lactobacillus casei* and *Lactobacillus brevis* are common beer spoilers (Asano et al., 2007). *Lactobacillus* phages belonging to the families *Siphoviridae* and*Myoviridae* have been isolated, while only a single *Lactobacillus* phage described thus far belongs to the *Podoviridae* family (Ackermann, 2007; Villion and Moineau, 2009). There is a relative paucity of genomic information regarding phages infecting members of this large and diverse genus, and there is limited taxonomic data regarding these phages (Mahony et al., 2012a). There are over distinct 100 species recognized within the *Lactobacillus* genus and with such host heterogeneity, it seems unsurprising that phages infecting species of this genus are equally complex and difficult to classify (Claesson et al., 2007). Currently, *Lactobacillus* phages are primarily classified based on the host species and subsequently into morphological or host range specific groups for a second tier of classification (For an extensive review of these phages, see Villion and Moineau, 2009). To date, the phage genomes of 24 *Lactobacillus* phages have been fully sequenced (http://www.ebi.ac.uk/genomes/phage.html) and their genetic complexity is clear with genome sizes ranging from ~31–42 kb. It is possible that with increased genome sequence data, identification of taxonomic groups for this diverse genus may be possible. *Lactobacillus* phages also exhibit morphological diversity and this characteristic may thus be used in their differentiation and taxonomy.

### Current limitations and future perspectives

Taxonomy of LAB-infecting phages has been the cornerstone of the development of detection and control tools, particularly pertaining to dairy fermentations. For example, several multiplex PCR systems have been established for the detection of lactococcal, S. *thermophiles*, and *Leuconostoc* phages (Labrie and Moineau, 2000; Del Rio et al., 2007, 2008; Ali et al., 2013). Such systems are essential to fermentation industries which rely on rapid identification of potentially problematic phages in order to limit phage proliferation within a plant. The practical relevance of phage taxonomy by far outweighs the apparent redundancy of repeated phage isolation, characterization and genomics studies as novel genetic elements, emerging phage species and evolving genome sequences continue to emerge. The vast information that currently exists for lactococcal phages has provided a solid basis for classification phages of LAB and other Gram positive bacteria. This data is based on more than three decades of isolation and characterization studies and genome sequencing efforts and have compounded the need for continual monitoring of phage populations. The loss of certain species (as single phage isolates may represent an entire species) and the identification of emerging and evolving phages present a significant challenge to phage taxonomy. With the exception of the Felix d'Herelle reference center for bacterial viruses in Canada, the general lack of centralized phage collection centers or the low uptake on requests for deposition of phage isolates in such collection centers is another issue that limits phage preservation and some phage isolates/species become obsolete if phage stocks are not maintained. Added to this is the lack of uniformity of classification methods. Classical studies relied upon serotyping and DNA-DNA hybridizations, which are time-consuming and not entirely discerning. In contrast, modern methodologies are becoming more reliant on genome sequencing, which has been possible through significant advances in sequencing technologies and throughput (Ronaghi et al., 1998; Eid et al., 2009; Meyer and Kircher, 2010). These advances together with the reduced cost of sequencing will be central to improving our knowledge of complex phage taxonomy groups, such as those represented by phages of the lactobacilli and those of underrepresented genera, including *Weisella*, *Oenococcus*, non-dairy *lactococci*, and *Leuconostoc* spp. It is evident that combinatorial strategies in phage taxonomy are still as useful today as they were in the past. Genomics combined with microscopic analysis is the current standard approach toward the classification of LAB phages with a decreased need for serotyping and exhaustive hybridization studies. One of the first attempts at unifying phage taxonomy was in 2002 by selecting a single structural protein (capsid or tail) as a phylogenetic marker and through this effort, *Siphoviridae* phages were classified into four groups (Proux et al., 2002). Following this, more sophisticated proteomic trees using overall proteomic data have been developed as a genome-based strategy for classifying phages. In 2002, the first phage proteomic tree of 105 phages was constructed. In this study, the *Siphoviridae* LAB-infecting phages clustered into one group of the proteomic tree, which may be sub-divided into the monophyletic taxonomic groups: sk1-like, TP901-1-like, Sfi21-like and the λ-like phages (Rohwer and Edwards, 2002). The *Siphoviridae* phages displayed most heterogeneity, while phages belonging to the remaining taxonomic groups (e.g., *Myoviridae* and *Podoviridae*) clustered into neat groups. This system places the LAB-infecting phages as part of the broader community of sequenced phages and such classification schemes are essential to understanding the overall relatedness and evolution of phages. In more recent years, this system has been expanded upon for *Myoviridae* and *Podoviridae* phages (Lavigne et al., 2006, 2009) and has endorsed the application of proteomics as a classification tool.

It is likely that LAB phage research will continue to focus on those phages that infect industrially significant genera as have been described above. It is also evident that the taxonomy of these phages requires regular review as the lessons learned from lactococcal phage taxonomy highlight the adaptive nature of phages in response to selective pressures in the industrial setting or the availability of alternative hosts (Fortier et al., 2006; Garneau et al., 2008). Therefore, phage taxonomy should be considered a fluid process that reflects the dynamic industrial environment which phages inhabit.

### Conflict of interest statement

The authors declare that the research was conducted in the absence of any commercial or financial relationships that could be construed as a potential conflict of interest.
